# High-Yield WS_2_ Synthesis through Sulfurization in Custom-Modified Atmospheric Pressure Chemical Vapor Deposition Reactor, Paving the Way for Selective NH_3_ Vapor Detection

**DOI:** 10.1021/acsami.4c10077

**Published:** 2024-09-02

**Authors:** Shuja Bashir Malik, Fatima Ezahra Annanouch, Ransell D′Souza, Carla Bittencourt, Milica Todorović, Eduard Llobet

**Affiliations:** † Universitat Rovira i Virgili, MINOS, Països Catalans 26, Tarragona Catalunya, 43007, Spain; ‡ IU-RESCAT, Research Institute in Sustainability, Climatic Change and Energy Transition, Universitat Rovira i Virgili, Joanot Martorell 15, 43480 Vila-seca, Spain; § TecnATox - Centre for Environmental, Food and Toxicological Technology, Universitat Rovira i Virgili, Avda. Països Catalans 26, 43007 Tarragona, Spain; ∥ Chimie des Interactions Plasma-Surface (ChIPS), Research Institute for Materials Science and Engineering, University of Mons, 7000 Mons, Belgium; ⊥ Department of Mechanical and Materials Engineering, Faculty of Technology, University of Turku, Vesilinnantie 5, 20500 Turku, Finland

**Keywords:** WS_2_, APCVD, sulfurization, gas sensor, NH_3_, 2D materials, TMDs, DFT

## Abstract

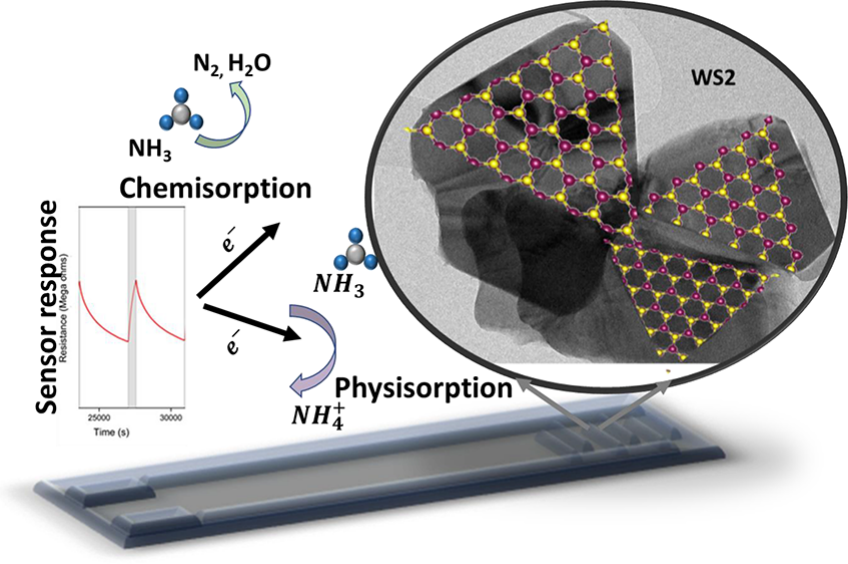

Nanostructured transition metal dichalcogenides have garnered significant research interest for physical and chemical sensing applications due to their unique crystal structure and large effective surface area. However, the high-yield synthesis of these materials on different substrates and in nanostructured films remains a challenge that hinders their real-world applications. In this work, we demonstrate the synthesis of two-dimensional (2D) tungsten disulfide (WS_2_) sheets on a hundred-milligram scale by sulfurization of tungsten trioxide (WO_3_) powder in an atmospheric pressure chemical vapor deposition reactor. The as-synthesized WS_2_ powders can be formulated into inks and deposited on a broad range of substrates using techniques like screen or inkjet printing, spin-coating, drop-casting, or airbrushing. Structural, morphological, and chemical composition analysis confirm the successful synthesis of edge-enriched WS_2_ sheets. The sensing performance of the WS_2_ films prepared with the synthesized 2D material was evaluated for ammonia (NH_3_) detection at different operating temperatures. The results reveal exceptional gas sensing responses, with the sensors showing a 100% response toward 5 ppm of NH_3_ at 150 °C. The sensor detection limit was experimentally verified to be below 1 ppm of NH_3_ at 150 °C. Selectivity tests demonstrated the high selectivity of the edge-enriched WS_2_ films toward NH_3_ in the presence of interfering gases like CO, benzene, H_2,_ and NO_2_. Furthermore, the sensors displayed remarkable stability against high levels of humidity, with only a slight decrease in response from 100% in dry air to 93% in humid environments. Density functional theory and Bayesian optimization simulations were performed, and the theoretical results agree with the experimental findings, revealing that the interaction between gas molecules and WS_2_ is primarily based on physisorption.

## Introduction

The discovery of graphene in 2004 marked the onset of research on two-dimensional (2D) materials owing to its distinctive and excellent physical, chemical, and electronic properties.^1,2^ However, the zero energy band gap of graphene limits its electronic applications.^2^ The demand for materials with specific chemical and electronic properties directed researchers toward novel 2D materials known as transition metal dichalcogenides (TMDs). Indeed, they have garnered tremendous attention driven by their tunable band gap, high carrier mobility, and environment friendliness.^3^ They are characterized by their X-M-X (or MX_2_) structure, where a transition metal (M) is sandwiched in between two chalcogen atoms (X) with strong interlayer covalent bonds and weaker interplanar van der Waals bonds.^4^ Moreover, the absence of dangling bonds on the TMD surface helps maintain stability and pristine quality.^5^ Due to these excellent electronic properties, TMD materials such as MoS_2_, WS_2_, SnS_2_, GeSe, InSe, and WSe_2_ are extensively studied for gas sensing applications.^6^

Tungsten disulfide (WS_2_) is one of the most important members of the TMD family and has captured the close attention of the scientific community. It is characterized by layered hexagonal symmetry forming S–W–S structure with an interlayer distance of ∼0.65–1 nm.^4^ Bulk WS_2_ has an indirect band gap of 1.4 eV, which can be tuned to the direct band gap of 2 eV in the case of monolayer WS_2_.^7^ It exhibits ambipolar field modulation behavior,^8^ excellent thermal conductivity (∼142 W/mK),^9^ outstanding flexibility,^10^ theoretically predicted high in-plane carrier mobility of (>1000 cm^2^V^–1^) owing to the reduced effective mass of charge carriers and a high current modulation ratio.^11^ The inherent distinctive properties of WS_2_ make it a highly sought-after 2D material. There is an imminent requirement for an industrial-scale synthesis method to cater to the burgeoning demand of the electronics market.

Numerous techniques have been developed to grow WS_2,_ including microwave irradiation,^12^ solvothermal synthesis,^13^ magnetron sputtering,^14^ molecular beam epitaxy (MBE),^15^ micromechanical exfoliation,^16^ and chemical vapor deposition (CVD).^2^ Among these, atmospheric pressure chemical vapor deposition (APCVD) and metal–organic chemical vapor deposition (MOCVD) appear as cost-effective techniques for achieving scalable, highly crystalline growth in micrometer scale^17^ to wafer scale.^18^ While wafer-scale synthesis of WS_2_ has been achieved using MOCVD, the use of organic compounds and reactive gases leads to a high possibility of carbon contamination,^19^ toxicity,^20^ and longer durations for large area synthesis^19,20^ of WS_2_. Also, precursor selection for MOCVD is complicated as it requires metal–organic compounds with high vapor pressure.^5^ On the other hand, precursor selection for APCVD is relatively straightforward, rendering the technique more technologically appealing. However, the high melting point of commonly used tungsten precursors such as WO_3_ (∼1473 °C) limits the growth of WS_2_ via APCVD and hinders the use of a wide range of substrates, including MEMS and flexible materials. Nonetheless, research has addressed this issue and reported the synthesis of WS_2_ at reduced temperatures using alkali halides.^17,21^ Li et al.^22^ successfully synthesized monolayer WSe_2_ and WS_2_ crystals within a temperature range of ∼700–850 °C using alkali halides like KCl, NaCl, KI, and KBr. The introduction of alkali halides decreased the sublimation temperature of the metal oxide precursor by forming volatile tungsten oxyhalides. Consequently, the growth temperature was found to be dependent on the melting temperature of the salt used. Furthermore, Shi et al.^17^ reported a correlation between the size of the as-grown WS_2_ crystals and the weight/weight (w/w) ratio of NaCl to WO_3_ in their NaCl-assisted APCVD process. This NaCl-assisted APCVD approach appears promising for the low-temperature synthesis of TMDs. However, there are still issues related to the synthesis process that deserve attention. Chang et al.^23^ reported that WS_2_ monolayers grown employing NaCl-assisted CVD undergo a degradation process, such as oxidation at random positions in the triangular monolayers. Additionally, the specific roles of the alkali metals and halide components are not fully understood in the CVD growth process.^24^ Moreover, various research groups have demonstrated alkali halide-free TMD growth via hydrogen-assisted CVD.^25^ However, the introduction of hydrogen in the growth process is not straightforward, as it can be a source of safety issues. Also, the growth of the triangular WS_2_ is often discontinuous, which may hinder their integration into real-world electronic applications.

Based on the literature, most studies have focused on directly growing WS_2_ on silicon oxide substrates. Consequently, the resulting films were often discontinuous with large triangles in the 2D plane orientation, which poses challenges for chemo-resistive gas sensor applications. Moreover, monolayer WS_2_ possesses a lower surface area compared to nanostructured multilayer WS_2_, which is advantageous for gas-sensing applications.^26^ It has been reported that edge-enriched, vertically oriented TMDs exhibit excellent gas-sensing properties.^27,28^ However, the synthesis of such out-of-plane oriented TMDs presents a significant challenge. The complex process of growing gas-sensitive TMD materials demands innovative and precision-based synthesis techniques. One of the main obstacles lies in achieving uniform, reproducible, and continuous deposition.^29,30^ To overcome these challenges, Alagh et al.^31^ reported hydrogen and alkali halide-free direct growth of continuous and vertically oriented WS_2_ nanotriangles on standard ceramic transducers for use in chemoresistive sensors. The authors employed a two-step CVD process by combining aerosol-assisted CVD (AACVD) and APCVD for the direct growth of WS_2_ onto transducer substrates at high temperatures (∼900 °C), limiting the use of the material for further applications, especially on flexible substrates. While the technique is promising for developing gas sensors at the laboratory scale, it may not be scaled up to the industrial level.

Considering the limitations of directly growing WS_2_ on substrates, we present, for the first time, a novel methodology for synthesizing sheet-like, edge-enriched WS_2_ powder on a scale of hundreds of milligrams per synthesis. This is achieved using a tube-in-a-tube arrangement with a hydrogen and alkali-halide-free APCVD technique. The large yield of this technique makes it scalable for mass production. It opens a new pathway toward developing ink formulations in the solvents of choice, such as inkjet printed or airbrushed onto a broad range of application substrates, including ceramic, silicon-based, or flexible polymeric materials. The morphology, phase composition, microstructure, and chemical composition of the synthesized WS_2_ powder were thoroughly investigated. Moreover, the growth mechanism and morphological evolution of the sheet-like WS_2_ were discussed. Additionally, gas-sensing films made from the synthesized WS_2_ powder were deposited onto alumina transducing substrates via a custom-built airbrush system. These films were studied toward low concentrations of NH_3_ at various operating temperatures (i.e., RT, 100 °C, and 150 °C) under dry and humid atmospheres. To gain insight into the atomistic mechanisms of interaction between NH_3_ and the sensing substrate, we employed density functional theory (DFT) simulations. The structure search for the optimal molecular adsorption configuration was accelerated by Bayesian optimization. This allowed us to compute the adsorption energy of NH_3_ on WS_2_ and examine the electronic structure of the adsorbate to determine the nature of the interaction. Finally, a gas-sensing mechanism is proposed in light of the experimental and DFT findings.

## Experimental Section

### APCVD Synthesis of Sheetlike WS_2_

Herein, a modified tube-in-tube APCVD technique was employed for synthesizing edge-enriched WS_2_ with a yield of hundred milligrams and possible scalability using a novel synthesis strategy. To do this, commercially available tungsten trioxide powder purchased from Sigma-Aldrich (CAS: 1314-35-8) was used and sulfurized to obtain edge-enriched WS_2_ nanostructures in a powder form. The sulfurization reaction was carried out at 900 °C for 60 min using metallic sulfur (Sigma-Aldrich, CAS: 7704-34-9) in a homemade tube-in-tube setup with a temperature gradient. The schematic of the setup is demonstrated in Figure S1. The middle zone of the furnace reaches the set temperature of 900 °C, as shown in the bright red colored heater coil in the schematic, Figure S1a,b. Zones adjacent to the middle zone of the furnace are at lower temperatures (approximately 400 °C) compared to the middle zone of the reactor. The temperature gradient of the furnace was checked with the help of a thermocouple. Three corundum boats were positioned within different temperature zones of the furnace; one boat contained 100 mg of WO_3_ precursor powder, while the other two boats contained 1 g of sulfur each. The boat containing WO_3_ was placed adjacent to a sulfur-containing boat inside a semisealed secondary quartz tube, both in the 900 °C temperature zone. The boat outside the secondary quartz tube is positioned upstream of the argon flow inside the bigger quartz tube. Before the sulfurization process, the quartz tube was flushed with 100 mL/min of Ar to remove any traces of oxygen in the reactor. The Ar flow was kept at 30 mL/min during the reaction. The furnace is programmed to heat from room temperature to the set temperature of 900 °C with a ramp of 40 °C/min. As soon as the furnace reaches 900 °C, the external quartz tube is carefully positioned such that the sulfur boat initially located outside the furnace is in the 400 °C temperature zone of the furnace, Figure S1b. This strategic placement of the boats facilitates the creation of a sulfur-rich environment, ensuring the sulfurization of WO_3_. The argon flow rate, ramp-up temperature, reaction time, and sulfur amount are essential parameters to be considered. The parameters were adjusted based on an optimization carried out in our previously reported work.^31^ The furnace was left to cool naturally after the reaction was completed. The synthesized WS_2_ powder weighed 110 mg.

### Gas Sensor Fabrication

WS_2_ sensing films were deposited, via a homemade airbrush system (Figure S2), onto commercial alumina transducer substrates (Ceram Tech GmBH, Germany), which have Pt-interdigitated electrodes with a gap of 300 μm on the front side and Pt-resistive heater meander on the back side. The electrode area was 2.5 mm × 5.1 mm. Before coating with WS_2_, substrates were cleaned by sequential sonication in acetone, ethanol, and deionized water, followed by blow drying with nitrogen. After that, the cleaned substrates were placed on the hot plate of our homemade airbrushing system, which was composed of a commercial airbrush, a hot plate, a multimeter, and connectors. Ten mg of WS_2_ powder was sonicated in 10 mL of absolute ethanol (Scharlab, CAS: 64-17-5) for 1 h to yield a brownish suspension. It is worth noting that the airbrushing technique allows us to use the solvent of choice. The prolonged sonication helps in exfoliating the WS_2_ but does not impact the morphology of the material. Due to its low boiling point, absolute ethanol was used to keep the thin film deposition temperature to the minimum possible value. Next, the obtained solution (3.5 mL) was transferred to the airbrush container, the electrodes of the substrate transducers were connected to the multimeter to control the resistance of the deposited films, and the hot plate was turned on and set at 55 °C. Finally, the brown solution was airbrushed onto the alumina substrates using N_2_ gas as a carrier. It is worth mentioning that this technique allows us to deposit thin films of the functional material over virtually any type of application substrate. The WS_2_ airbrushing deposition process was tested on poly(methyl methacrylate) (PMMA), Kapton, silicon, and glass substrates to evaluate WS_2_- film durability and deposition efficacy. Results demonstrated excellent adhesion and film quality across all substrates, underscoring the versatility of the airbrushing technique, Figure S3. Additionally, we conducted more than one hundred bending tests, subjected the deposited films to high-pressure air flows, and performed water droplet tests to evaluate the durability of the films. This comprehensive assessment explored film durability and affirmed the efficacy of airbrushing across diverse material surfaces.

### Material Characterization Techniques

The sheets-like edge enriched WS_2_ nanostructured morphology was examined using a field emission scanning electron microscope (Thermo Scientific Scios 2). High-resolution transmission electron microscopy (HRTEM) studies were carried out using a JEOL F200 TEM ColdFEG operated at 200 kV. WS_2_ powder was dispersed in absolute ethanol using sonication and drop cast onto carbon-coated copper grids for TEM and HRTEM analysis. TEM images were acquired with a Gatan OneView camera, a CMOS-based and optical fiber-coupled detector of 4096 by 4096 pixels. X-ray diffraction (XRD) was used to analyze the crystal structure of the synthesized material. The XRD measurements were made using a Bruker-AXS D8-Discover diffractometer equipped with a parallel incident beam (Göbel mirror), vertical θ–θ goniometer, XYZ motorized stage, and a GADDS (general area diffraction system). The X-ray diffractometer was operated at 40 kV and 40 mA to generate Cukα radiation. The GADDS detector was a VÅNTEC-500 (silicon strip technology) placed 15 cm from the sample. The Raman spectra were obtained using a Renishaw in Via, laser 514 nm, ion argon – Novatech, 25 mW. For XPS measurements, a Versaprobe PHI 5,000 from Physical Electronics was used to record the spectra. The X-ray source Al Kα was monochromatized, and measurements were taken at a takeoff angle of 45° from the sample surface. The spot size of 200 μm was utilized, and a pass energy (PE) of 20 eV was employed for the spectra recorded in the core level binding energy regions: W 4f, S 2p, and C 1s. To counter charge build-up on the sample surface during measurements, a dual beam charge neutralization consisting of an electron gun (<1 eV) and an argon ion gun (<10 eV) was implemented. The XPS spectra were examined using CASA-XPS software.

### Gas Sensing Measurements

The gas sensing measurements of the as-fabricated WS_2_ sensors were performed using a homemade gas detection system employing a Teflon chamber with a volume of 35 mL. The chamber can accommodate four sensors simultaneously. The chamber consisted of an inlet connected to the gas delivery system and an outlet connected to an exhaust. The chamber was connected to a fully automated gas flow measurement setup with the ability to supply diluted gas mixtures via mass flow controllers (Bronkhorst High-Tech B.V.). Calibrated gas cylinders balanced in dry synthetic air (Air Premier purity: 99.999%) were used for gas sensing measurements. The operating temperatures of the sensors were controlled by connecting the meander heaters of the sensors to an external power supply (Agilent U8002A). Sensor responses were recorded using an Agilent-34972A data acquisition system by monitoring the sensing material resistances upon exposure to different concentrations of target gases such as NH_3_, NO_2_, H_2_, CO, and benzene. Sensors were operated at room temperature (RT), 100 and 150 °C. A continuous dry airflow of 100 mL/min was maintained in the chamber for 3 h before initiating gas sensing measurements to ensure baseline stabilization. The sensors were exposed to the target gas for 10 min, followed by exposure to dry air to recover and stabilize the baseline. The baseline recovery time was adapted according to the sensor operating temperature: 60 min for 100 and 150 °C and, for room temperature operation 120 min. A 100 mL/min overall flow rate was maintained throughout the gas sensing measurements. For a reducing species like NH_3_, the sensor response was calculated using eq 1, while for oxidizing species like NO_2_, the relative response was calculated using eq 2.
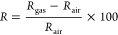
1
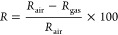
2*R*_air_ and *R*_gas_ are the real-time resistances of sensors when exposed to air and to target gas, respectively.

### Computational Methods

#### First-Principles Calculations

all simulations were performed using density functional theory (DFT). We utilized the all-electron, numeric atom-centered orbital code FHI-aims,^32^ incorporating the Perdew–Burke–Ernzerhof (PBE) exchange–correlation functional^33^ and Tkatchenko–Scheffler (TS) vdW corrections.^34^ To ensure convergence, the DFT calculations employed tier-1 basis sets, light grid settings, and a 4 × 4 × 1 Monkhorst–Pack k-point grid. The total energy was converged within 10–6 eV, and the structures were optimized until all force components were reduced below 10–5 eV/Å. To achieve a comprehensive description of the electronic structure and to incorporate the effects of spin–orbit coupling, we utilized relativistic corrections and explicitly included spin–orbit coupling terms in our calculations.^35^ A vacuum spacing of 22 Å was employed along the *z*-direction to mitigate interactions between the substrate layers. To prevent interactions between adsorbed NH_3_ molecules, we adopted a 4 × 4 x-y hexagonal supercell of the WS_2_ substrate (lattice constant a = 12.7 Å), resulting in a distance of over 10 Å between molecule images in neighboring periodic cells. The combined system comprised a total of 52 atoms, including four atoms for the NH_3_ molecule and 48 WS_2_ substrate atoms. The single-trilayer thick WS_2_ substrate was modeled using a hexagonal unit cell characterized by an optimized lattice constant of 3.168 Å, a value that closely aligns with experimental^36^ and previous computational findings.^37^ The adsorption energy between the substrate and the target molecule (*E*_ads_) was evaluated as the difference between the total energy of the adsorbed system *E*_tot_) and the sum of the isolated energies of the substrate (*E*_WS_2__) and the molecule (*E*_NH_3__):
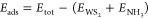
3

#### Adsorbate Structure Identification

the identification of stable ammonia adsorbates on WS_2_ was achieved through the application of DFT within the “Bayesian optimization structure search” (BOSS) code.^38^ BOSS, a machine learning-driven approach, accelerates the identification of energetically favorable structures by strategically navigating the adsorption energy surface (AES). Given an initial data set of configurations, BOSS constructs the most probable surrogate model of the AES by Gaussian process regression. This surrogate model is then iteratively refined through an active learning process, enabling the identification of energetically stable adsorbates within the AES minima, as demonstrated in previous adsorption studies.^39^

The structures of the molecule and substrate were optimized independently, then fixed and deployed as “building blocks” during the structure search. The adsorption energy surface (AES) was explored in a 6D search space as a function of molecular position and orientation above the substrate. In practice, we applied three translational degrees of freedom (*x*, *y*, *z*) and three rotational degrees of freedom (α, β, γ) to the molecular center of mass (centroid) to perform configurational sampling. The rotational degrees of freedom for α and β were confined within the range of 0 to 360°, whereas that of γ was restricted to the range of 0° to 120°, owing to the rotational symmetry of the NH_3_ molecule along the *z*-axis. The rotational angles (α, β, γ) were implemented around the internal molecular frame of reference, as defined in Figure S4a. The translations were applied in the directions of crystallographic axes [100], [010], and [001]. Because of the periodic nature of the substrate model, the x-y search was confined to a limited section of the substrate, as depicted in Figure S4 (yellow lines). Initial tests indicated that the optimal bounds for the molecule height z were between 2 and 2.8 Å computed from the highest surface atom. Adsorption energetics were sampled with the exploratory Lower Confidence Bound (eLCB) acquisition function,^40^ which strikes a balance between exploitation and exploration. To build our 6D kernel, we applied the standard periodic kernel to describe all the periodic degrees of freedom except the z coordinate, where we used the radial basis function kernel. The 6D BOSS search was initialized with 200 Sobol space-filling points and continued during 2000 active learning iterations. After we observed the convergence of the global minimum of adsorption energy, we identified the atomic configuration behind the inferred global minimum and fully optimized the geometry.

#### Electronic Transport and Sensor Resistivity

given the atomic coordinates for the global minimum structure, the Boltzmann transport equations (BTE) were employed to calculate sensor resistivity. Here, the band structure extracted from the FHI-aims calculations provided crucial inputs for calculating transport properties with the Boltztrap2 code.^41^ The BTE enables the evaluation of electrical conductivity along two orthogonal principal axes ([100] and [010]) within the *x*–*y* plane of the two-dimensional WS_2_ layer. The calculated conductivity is averaged over these principal directions. Within the framework of the Boltzmann transport approximation, the electrical conductivity is expressed as follows:
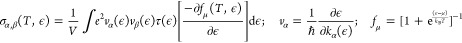
4Here, *v*_α_ is the energy (ϵ)-dependent group velocity of the band electrons along the component, *e* is the electrical charge, *V* is the unit cell volume of the WS_2_/NH_3_ system, τ(ϵ) is the energy-dependent relaxation time, *T* is the temperature, μ is the chemical potential, *f* is the Fermi–Dirac distribution, *k*_*B*_ is the Boltzmann constant, and ℏ is Planck's constant. The chemical potential associated with the experimentally applied gate voltage^42^ was determined by fixing its value as a fitting parameter to the experimental baseline resistivity curve of WS_2_ at 0.43 eV in the absence of the target molecule, NH_3_.

The resistivity was then defined as the reciprocal of conductivity, and the sensor sensitivity was calculated using eq 1. The calculated sensor response function was obtained by solving the “step response differential equation”,
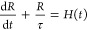
5where “*t*” is the time, “*R*” is the time-dependent resistivity, and *H*(*t*) is the Heaviside step function. The initial conditions for the resistivity during the “gas-in” phase were set as *R*_initial_ = *R*_WS_2__ and *R*_final_ = *R*_WS_2_+NH_3__. The initial conditions were reversed for the “gas-out” phase. The characteristic time (τ) is a function of the adsorption energy and temperature. τ is also known as the time constant since the differential equation is solved at a constant temperature.

## Results

### Characterization of the Synthesized WS_2_ Powder

**Figure 1:**
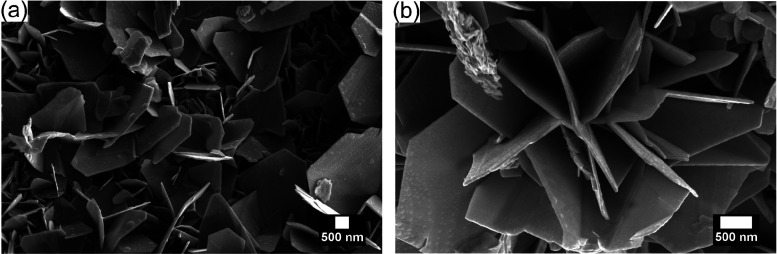
FESEM images depicting (a) triangle-like structures of WS_2_ and (b) agglomeration of the triangles in the form of flower-like structure.

Figure 1 shows FESEM micrographs for the as-synthesized WS_2_. The analysis of the FESEM images reveals that WS_2_ shows a distinctive zigzag-edged triangular sheet architecture with an average thickness of 36 nm. Notably, the majority of the triangle-like nanostructure exhibits an edge-to-edge width of 255 nm, as presented in Figure 1a and Figure S5. In comparison, some of the triangles show a higher edge-to-edge width of 690 nm. The size of the synthesized material was calculated using the inbuilt feature of Scios2 FESEM equipment and ImageJ software. Interestingly, these nanostructures can also be self-assembled in the shape of flowers, as can be seen in Figure 1b. The tendency of WS_2_ to grow in triangularly shaped layered structures has been demonstrated earlier as well.^43−45^ The reason behind the triangular architecture of the WS_2_ grown via the CVD technique may be owed to the growth rate of different crystal planes of S atoms or W atoms and also to the growth temperature.^46^ This has been confirmed in the growth mechanism of MoS_2_.^47^

**Figure 2:**
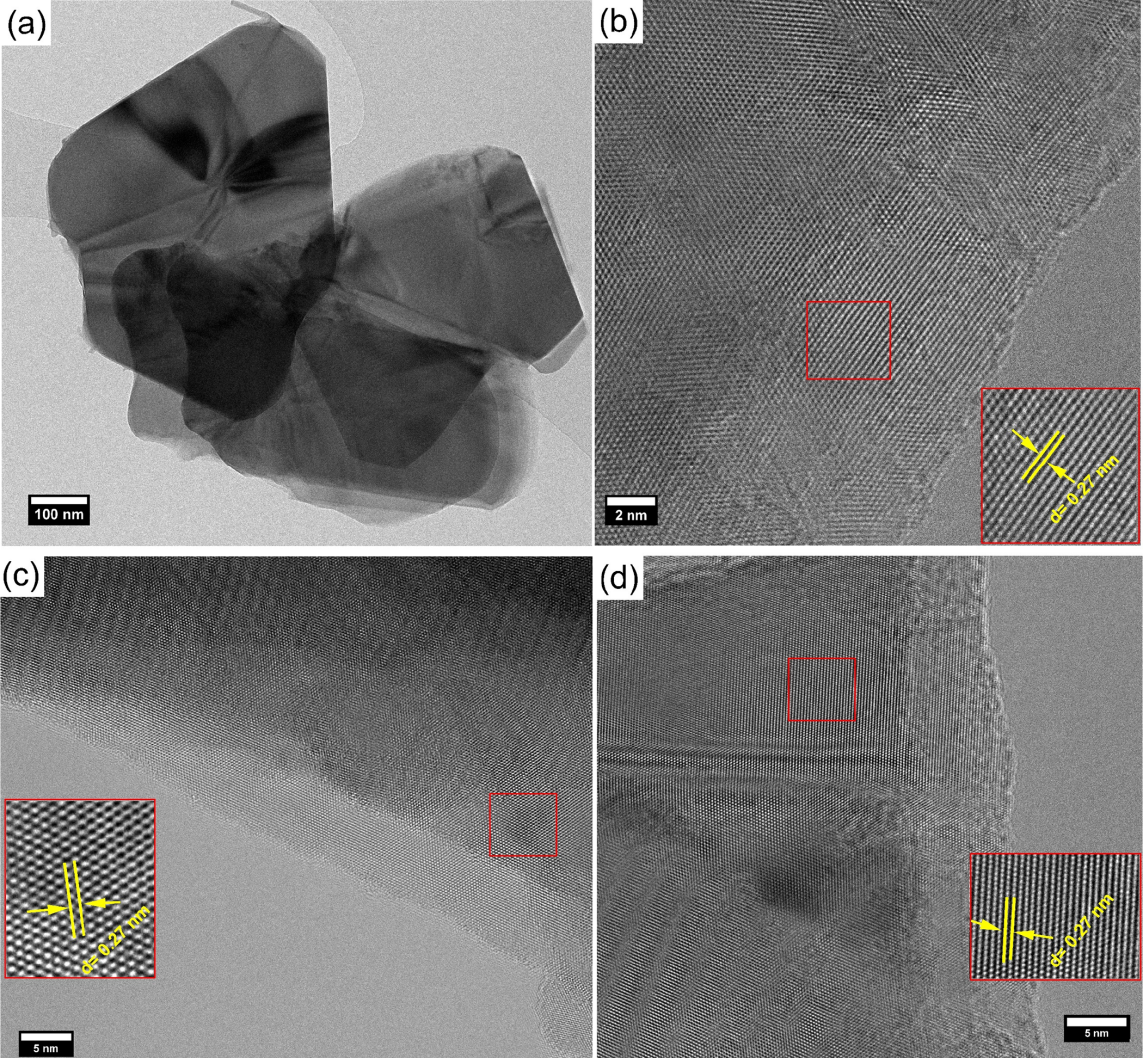
(a) TEM image of WS_2_, (b–d) HRTEM images of WS_2_.

TEM analysis was employed to examine the morphology of the as-synthesized material, Figure 2. From panel (a), we can observe the presence of large, thin, and transparent triangles, which is consistent with the results observed with FESEM. Indeed, tungsten trioxide powder has completely transformed into 2D triangle-like WS_2_ structures. The length of the edge sides ranged from 250 to 690 nm. This agrees with the FESEM results. High-resolution (HR) TEM image is displayed in panels (b–d) with insets. It allows us to evaluate the composition and the crystallinity of the triangle-like structures. From these images, we can conclude that each triangle is composed of multilayer WS_2_ material; the layers were stacked one on top of the other with interplanar spacing (d) of 0.27 nm corresponding to the (101) plane of WS_2_ (ICDD card number: 84–1399). This was confirmed by XRD as well.

**Figure 3:**
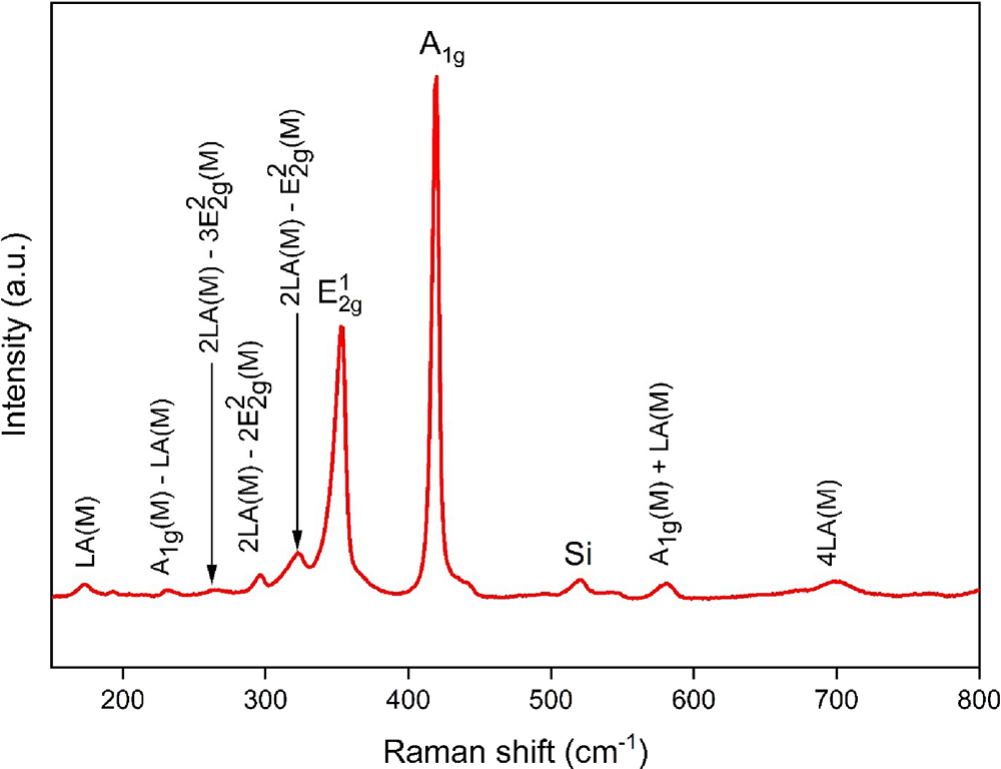
Raman spectrum of as-synthesized WS_2_.

The crystallographic structure and the purity of the synthesized WS_2_ powder were evaluated by X-ray diffraction (XRD). Figure S6 depicts the XRD diffractogram of as-synthesized WS_2_ airbrushed on a silicon oxide substrate. The detailed results are presented in the Supporting Information. Raman spectroscopy is an acknowledged powerful and nondestructive characterization technique to study the purity of layered materials such as transition metal dichalcogenides. In this respect, Raman spectroscopy was used to study the as-synthesized WS_2_ using a 514 nm wavelength laser. Figure 3 displays the recorded Raman spectrum. The results show two intense peaks at 352 and 418 cm^–1^, which correspond to in-plane vibrational *E*_2g_^1^ (Γ) and out-of-plane A_1g_(Γ) modes, respectively.^48^ The residual peaks found in the spectrum can be identified as longitudinal acoustic phonons (M), A_1g_(M) – LA(M), 2LA(M) – 3E_2g_^2^(M), 2LA(M) – 2E_2g_^2^(M), 2LA(M) – E_2g_^2^(M), A_1g_ + LA(M), and 4LA(M) modes of WS_2_.^46,49^ The longitudinal acoustic phonons are in-plane collective movements of atoms in a lattice. The ratio of peak intensity I[E_2g_^1^]/I[A_1g_] comes out to be 0.84, indicating the formation of multilayer WS_2_,^50^ consistent with HRTEM results. No peaks corresponding to WO_3_ were found in the Raman spectrum. While Raman spectroscopy did not detect any peaks corresponding to WO_3_, indicating a predominant presence of WS_2_, XRD and XPS analyses revealed some residual WO_3_ detected. XPS quantified this as 14% of tungsten atoms remaining as WO_3_.

**Figure 4:**
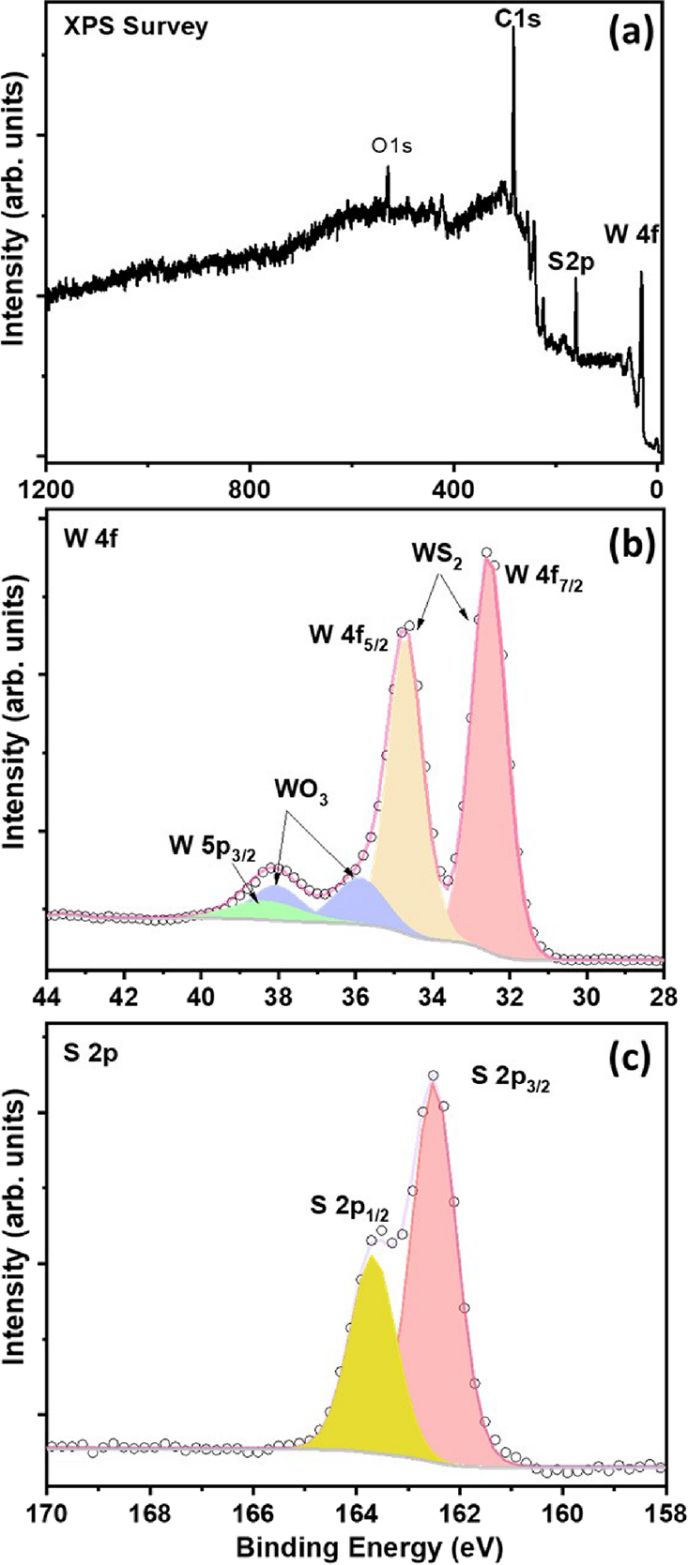
XPS spectra of as-synthesized WS_2_: (a) XPS survey, (b) W 4f core level, and (c) S 2p core level.

X-ray photoelectron spectroscopy was used to explore the chemical composition of the deposited samples. The XPS survey spectrum shows the presence of W, C, S, and O (Figure 4a). The spectra recorded in the core-level regions allow for determining the oxidation state of the elements. The spectrum recorded in the W4 f binding energy region is well reproduced by one singlet centered at binding energy 38.4 eV corresponding to the W 5p_3/2_ core level in WS_2_ and two doublets. The doublet with W4 f7/2 peak centered at 32.5 eV corresponds to tungsten atoms in the (4+) valence state in WS_2_, while the one at 35.9 eV, to tungsten atoms in the (6+) valence state in WO_3_ (Figure 4b). A detailed analysis of this spectrum results in 86% of tungsten atoms participating in W–S bonding in WS_2_ and 14% in WO_3_.^31^ The S 2p spectrum, known by the doublet peaks, S 2p_1/2_ and S 2p_3/2_ at 163.7 and 162.5 eV, respectively, with a spin–orbit energy separation of 1.2 eV corresponding to WS_2_ (S2– oxidation state), can be seen in Figure 4c. Additionally, the S 2p spectrum of the examined samples does not exhibit any discernible S–O bond component at 168.8 eV.^31,51^ Therefore, the obtained spectra confirm the formation of WS_2_ with the presence of a small amount of WO_3_ impurities.

### WS_2_ Synthesis Mechanism

Sulfurization of WO_3_ is an interesting and intriguing process. WO_3_ and S powders are the main precursors predominantly used for WS_2_ growth by CVD.^52^ WO_3_ is partially reduced in the sulfur vapor environment to form suboxide species, WO_3–*x*_, which is further sulfurized to form WS_2_.^53^ The reaction involved is
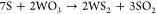
6

Keeping suboxide species into consideration, the growth stages of WS_2_ could be mapped onto two hypothesized reaction stages as proposed by Li and Li^54^ and Ji et al.^55^ The reactions proposed are
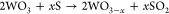
7
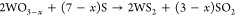
8

This is a complex conversion from monoclinic WO_3_ to hexagonal cells of WS_2_ since the W atom sites in monoclinic WO_3_ cells differ significantly from those of hexagonal unit cells of WS_2_. This indicates that the W–W interatomic distances along a and c axes change from 0.73 nm in WO_3_ to 0.3158 nm in WS_2_ along the *a* axis and from 0.767 nm in WO_3_ to 1.8 nm in WS_2_ along the *c* axis.^56^ The lattice parameters of the synthesized WS_2_ and WO_3_ precursor are discussed and demonstrated in the XRD characterization section in the Supporting Information. WO_3–*x*_ and S react heterogeneously in the gas phase and grow laterally. This process forms randomly distributed flakes rather than a continuous film.^57^

In this work, the sulfur powder placed in the boat within the secondary tube starts to sublimate above 100 °C, Figure S1a. The sublimated sulfur is carried by Ar gas flow toward the WO_3_ precursor powder boat to maintain the subsequent sulfur-rich environment in the growth zone. Since the sulfurization reaction is carried out in a hydrogen-free environment, the reduction of WO_3_ is comparatively slower. Hence, the sulfurization reaction is continued by introducing more sulfur vapors into the reaction chamber, Figure S1b. The growth mechanism schematic of the edge-enriched WS_2_ plates is depicted in Figure S7.

### Gas Sensing

The gas sensing properties of the as-fabricated WS_2_ sensors were tested for ammonia gas and evaluated using a homemade gas monitoring system. To assess the optimal working temperature, the sensors were studied toward 5 ppm of NH_3_ at room temperature- RT, 100 °C, and 150 °C. Overall, operating temperature is a crucial factor in gauging sensor performance; this is because the sensitivity, selectivity, and response/recovery dynamics of gas sensing materials heavily depend on operating temperature. A simple procedure to identify the optimum temperature is measuring a single gas concentration of the analyte gas at different operating temperatures. It is worth noting that the maximum operating temperature was set at 150 °C as above 150 °C the evaporation of the sulfur could potentially deteriorate the sensing material leading to the formation of WO_3_/WS_2_ complex.^31^ Also, operating devices at low temperatures is beneficial for developing low-power devices.^30,31^

**Figure 5:**
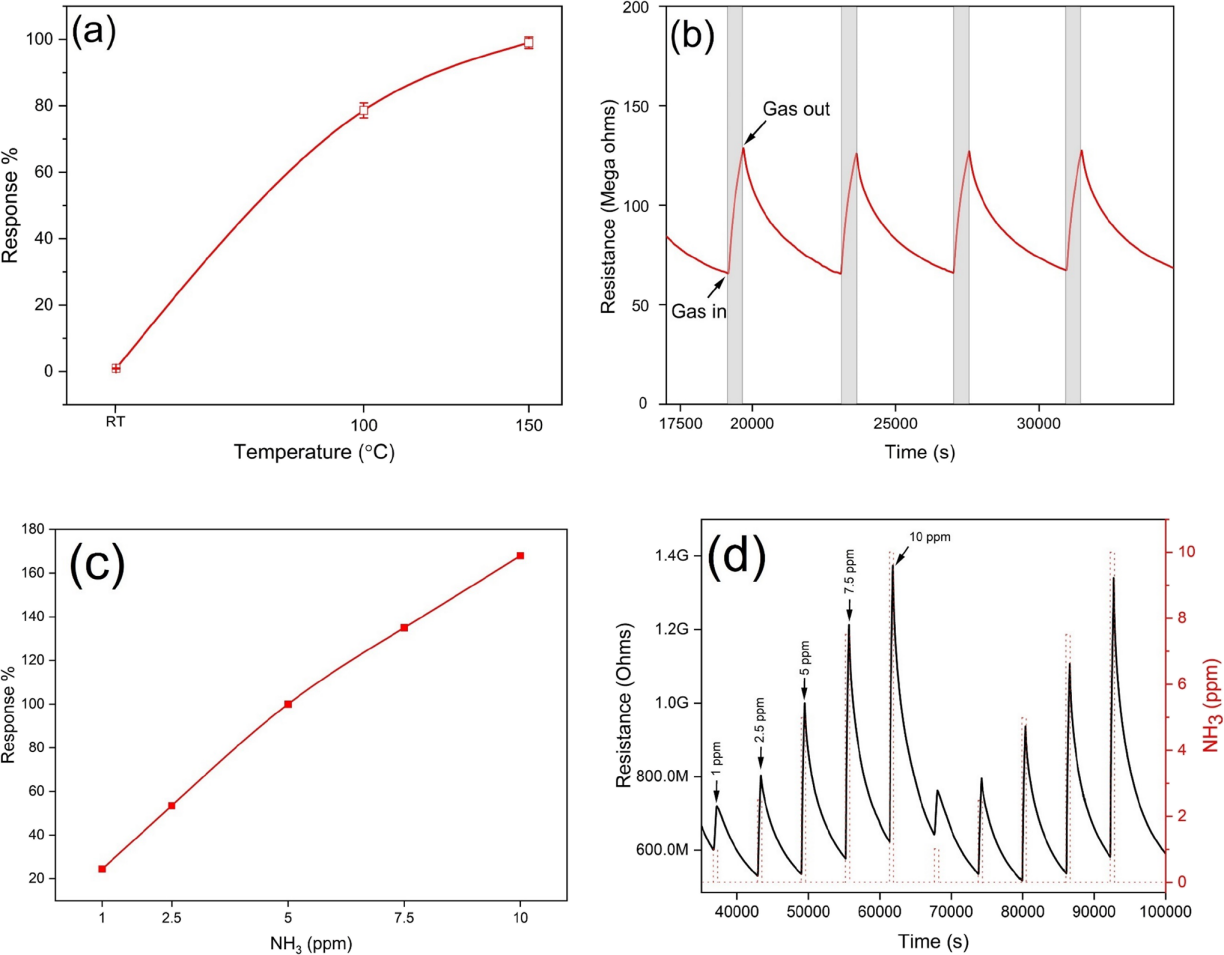
(a) Typical sensor response as a function of temperature toward 5 ppm of NH_3_, (b) sensing film resistance changes as a function of time toward 5 ppm of NH_3_ at 150 °C, (c) WS_2_ sensor response as a function of NH_3_ concentration at 150 °C, and (d) gas sensing film resistance changes as a function of time toward different NH_3_ concentrations at 150 °C.

Figure 5a shows the sensor responses toward 5 ppm of NH_3_ at different operating temperatures ranging from RT (25 °C) to 150 °C. As depicted in the figure, the sensor responses increase with an increase in the operating temperature. The standard deviation of the sensor responses is negligible, indicating highly reproducible and stable sensing characteristics. The maximum sensor response at high temperatures could be owed to the enhanced gas molecule adsorption. Indeed, with the increase in the temperature, the activation barrier layer is lowered, enhancing the rate of gas adsorption and leading to higher responses.^31^ Thus, the operating temperature for all the subsequent studies was established to be at 150 °C, which is quite low compared to the standard operating temperatures found in metal oxide-based gas sensors.^58^

Furthermore, the sensor responses at room temperature toward 5 ppm of NH_3_ were calculated to be 0.89%, rising to 80% at 100 °C and 100% at the optimal working temperature (150 °C). Figure 5b shows real film resistance changes as a function of time toward 5 ppm of NH_3_ at 150 °C. Upon being exposed to ammonia, a reducing gas species, the WS_2_ sensor responds as a p-type semiconductor, showing an increase in the resistance. In line with earlier NH_3_ studies^59,60^ after the adsorption of the NH_3_ molecules onto the WS_2_ sensing material, a charge transfer occurs. The NH_3_ molecules donate electrons to WS_2_ leading to an increase in the resistance. The sensors show stable and reproducible responses toward the target gas. The sensor baseline recovers well after each exposure cycle.

Moreover, the sensors were tested for a wide range of NH_3_ gas concentrations ranging from 1, 2.5, 5, 7.5, and 10 ppm at the optimal working temperature. The responses were calculated to be 24.45%, 53.44%, 100%, 135% and 168% respectively. As it is evident from Figure 5c, there is a linear increase in the response with the increase in the concentration of ammonia. The standard deviation error of the responses is so small that it can hardly be observed in the figure, indicating the stable and reproducible sensing responses of the sensors. As anticipated, increasing the concentration of NH_3_ leads to higher resistance changes in the WS_2_ sensor, resulting in enhanced responses. The linear trend of the sensor responses reveals that the sensors do not saturate by exposure to ammonia concentrations below 10 ppm. The dynamics of gas sensor film resistance change toward different ammonia concentrations is presented in Figure 5d. As is evident, the sensors can detect ammonia concentrations as low as 1 ppm. These concentrations are much lower than the legal airborne permissible exposure limit (PEL) of 50 ppm as recommended by the Occupational Safety and Health Administration (OSHA) over an 8 h work shift.^61^ Also, as per XPS and XRD results, there is residual WO_3_ present in the synthesized material. WO_3_ provides active sites for gas adsorption. WO_3_ is one of the best-suited materials for gas sensing, particularly for NO_2_. However, WO_3_ operates at comparatively higher temperatures (>200 °C). In contrast, the WS_2_-based sensors demonstrate excellent selective gas sensing properties at 150 °C. Therefore, the impact of the presence of WS_2_ is significant.

Selectivity is one of the most important criteria to determine the performance of a sensor. The selectivity of WS_2_ sensors was evaluated toward fixed concentrations of various interfering gases such as benzene, carbon monoxide (CO), nitrogen dioxide (NO_2_), and hydrogen (H_2_) at 150 °C—these analyte gases as particularly important to test for selectivity owing to their potential health and environmental risks. For example, NO_2_ is one of the main contributors to acid rain;^62^ hydrogen could be potentially dangerous for its highly explosive and flammable properties,^63^ and exposure to 5 ppm of benzene for more than 15 min has been linked to the development of cancer.^30^ The radar plot depicted in Figure S8 shows the responses of the sensors toward tested gases at 150 °C. The results show that among all the gases, the sensors respond toward NH_3_ and C_6_H_6_ only with the highest response toward NH_3_. These results suggest high selectivity toward NH_3_ gas. It is worth mentioning here that all the gas concentrations tested are way below the permitted limits.^30,64^ Also, compared with reported studies on WS_2_ and/or other TMDs or a composite of WS_2_, our sensors show better responses with a low experimental limit of detection (LoD) as can be seen in Table S1. While the research works listed in the table focused on ammonia concentrations beyond the permissible limits, it is worth noting that some studies reported favorable sensor responses. This drawback will limit the real applications of these sensors. In contrast, our sensors excel in detecting ammonia gas at levels below 1 ppm, demonstrating their remarkable sensitivity even in low-concentration environments.

The effect of ambient moisture dramatically impacts the gas sensor sensitivity by affecting the electrical properties of the sensing material. This makes it mandatory to evaluate the gas sensor performance under the presence of humidity and verify the sensor capability for real-world applications. Figure S9 illustrates the WS_2_ sensor responses toward 5 ppm of NH_3_ under dry and humid (50% relative humidity at 25 °C) environments. It is observed that the sensor response decreased slightly from 100 to 93%. The baseline resistance decreased from ∼60 MΩ in a dry environment to ∼50 MΩ in a humid environment. In general, during the gas sensing measurements in humid environments, there is competition between hydroxyl groups (water vapors) and ammonia gas molecules. Depending on the relative surface concentration of the hydroxyl groups, the impact of the humidity becomes noticeable.^29^ If the concentration of the oxygen species is lower than the surface concentration of the hydroxyl groups, the sensor response decreases. On the contrary, if the sensors demonstrate high moisture resistance, this signifies that most of the active sites are occupied by adsorbed oxygen species. This results in no or little change in the sensor performance. Hence, the edge-enriched WS_2_ sensors exhibit a high level of immunity against elevated moisture levels. This characteristic feature makes them highly suitable for real-world applications.

### Theoretical Modeling: DFT Adsorption Simulation Results

**Figure 6:**
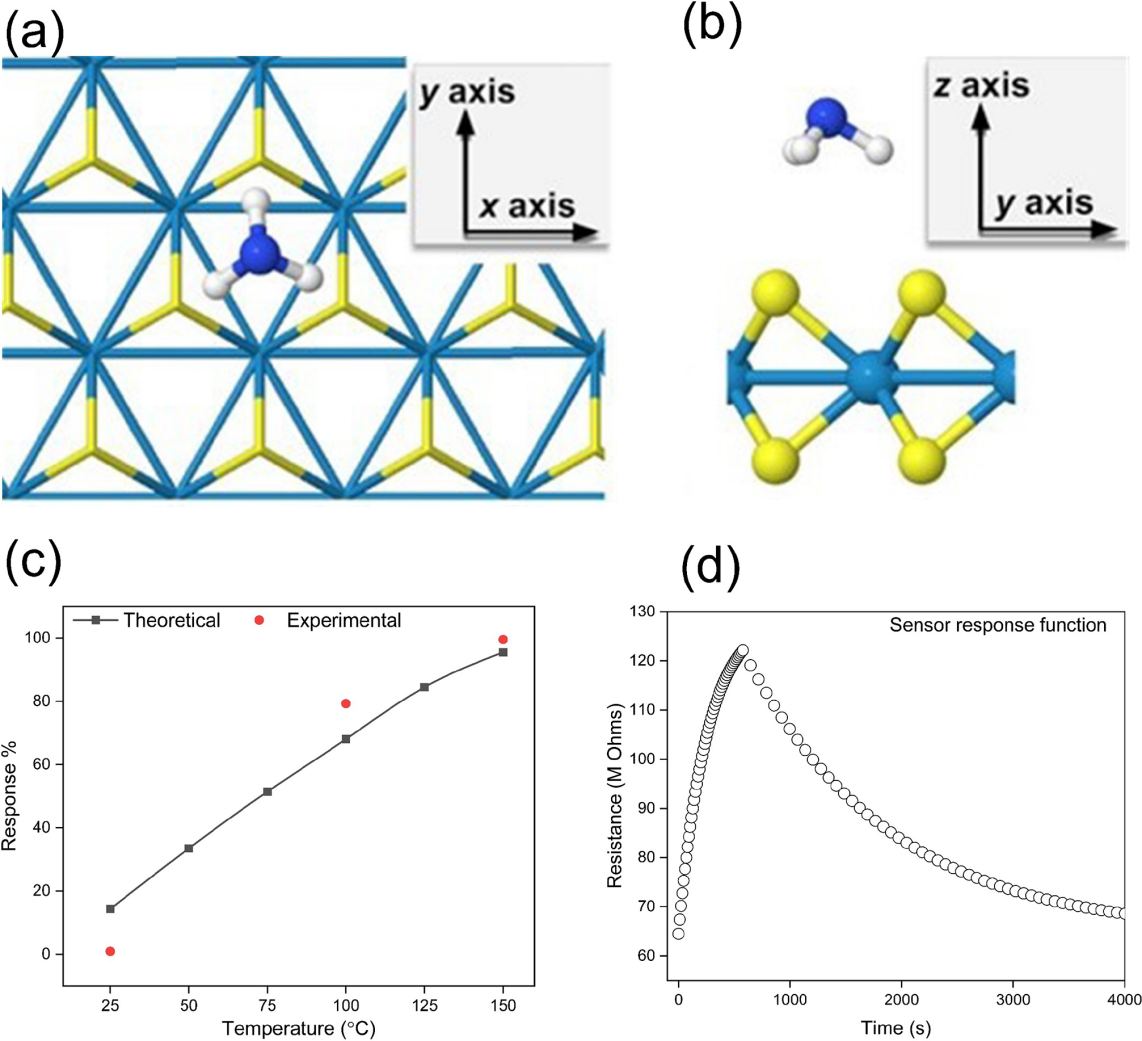
Computed adsorption configuration and transport properties. (a, b) Top and side view of the global minimum adsorbate structure of NH_3_ on WS_2_. (c) Temperature-dependent sensor sensitivity from RT to 150 °C. (d) sensor response function computed at 150 °C.

After a 6D Bayesian optimization structure search, the global minimum of the adsorption energy landscape was determined to be at coordinates *x* = 3.19 Å, *y* = 1.87 Å, *z* = 2.38 Å, and angles α = 0.0°, β = 179.31°, and γ = 85.15°. During the subsequent structural optimization, the structural and energetic changes were minimal. The global minimum energy of adsorption was 0.21 eV. After adsorption, the molecule was positioned at a height of 2.4 Å above the hollow surface site (the gap between adjacent S atoms), illustrated in Figure 6a. The three molecular H atoms were oriented toward the substrate and aligned in the direction of three S atoms in the uppermost layer of the substrate (see Figure 6b).

The Mulliken analysis of partial atomic charges was employed to compute the charge transfer between the WS_2_ structure and the NH_3_ target molecule. We observed a very small net charge transfer of 0.024*e* from the WS_2_ lattice toward the NH_3_ molecule (*e* denotes the elementary charge). To explore the nature of the chemical bond, we analyzed the partial density of states (DOS) of the WS_2_ substrate and NH_3_ molecule near the Fermi level. We compared them to the electronic states of the isolated gas molecule (not shown here). The results revealed that the electronic states of the molecule remained as narrow as in the gas state, with no evidence of hybridization upon surface adsorption. The calculated band gap of 1.78 eV for the adsorbed system was similar to the WS_2_ band gap of 1.64 eV. All the electronic structure observations suggest the absence of covalent bonding between the NH_3_ molecule and WS_2_ substrate and point to physisorption as the main mechanism of interaction.

For the adsorption configuration above, we computed the temperature-dependent resistivity, corresponding sensitivity, and sensor response. Figure 6c illustrates the rate at which sensor sensitivity increases as operating temperature rises beyond the room temperature conditions. Subsequent computational analysis demonstrated that the increasing trend is a direct result of molecular adsorption. For the bare WS_2_ substrate, we recorded a drop in sensitivity with increasing temperature, as expected for a semiconductor. Upon NH_3_ adsorption, the shift in the Fermi level was accompanied by a flattening in band curvature, which produced a notable decrease in charge carrier mobility and a jump in resistivity. Rising temperatures made this effect more expressed and resulted in increasing sensor sensitivity. This trend was in very good agreement with experimental data, as observed in Figure 6c. Next, we consider the sensor response function in Figure 6d. The time evolution of the sensor response was calculated at *T* = 150 °C to match the experimental conditions. This property is also in good agreement with the experimental observation in Figure 5b and Figure S9.

**Figure 7:**
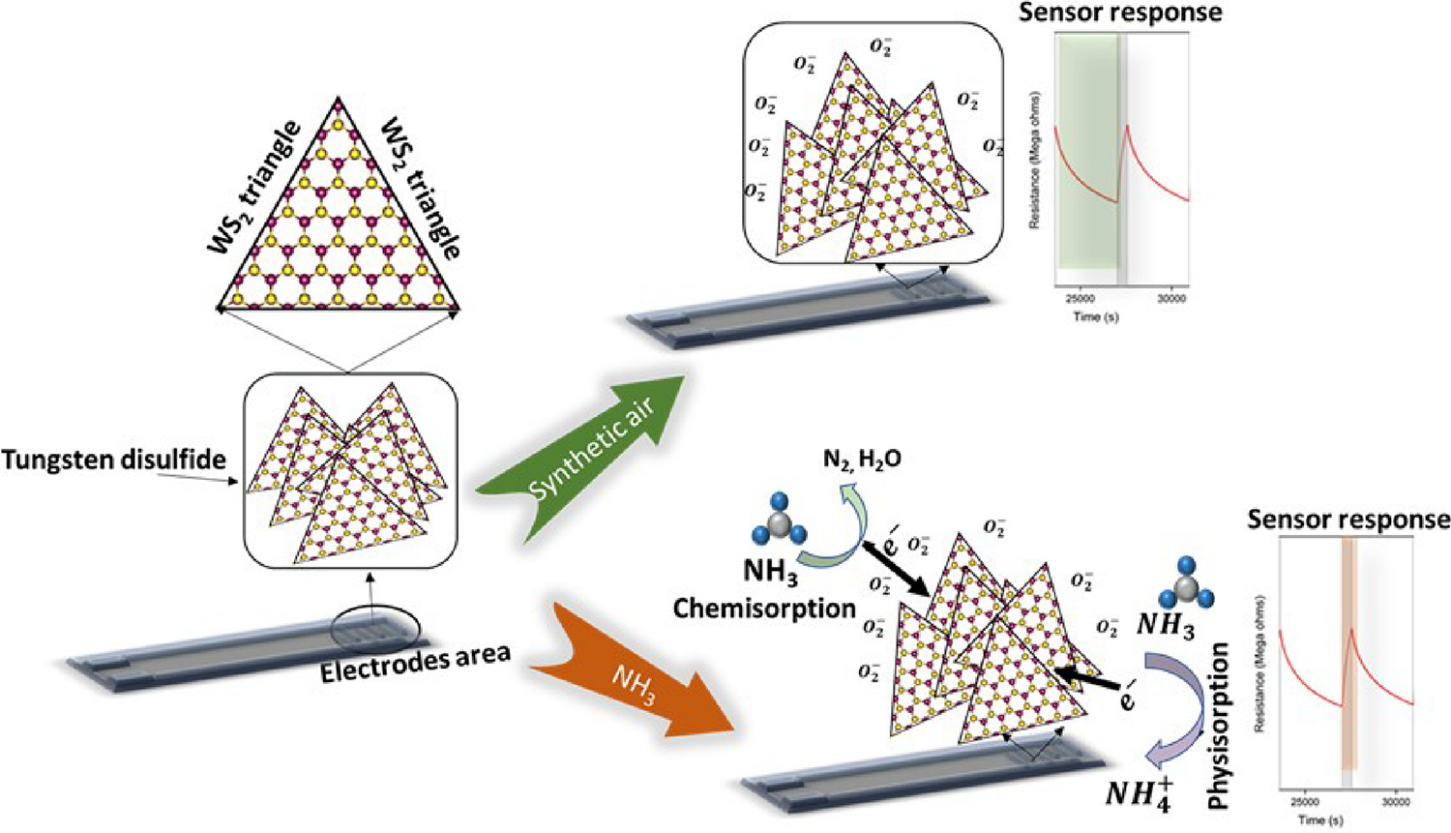
Proposed gas sensing mechanism.

Based on the obtained results, the gas sensing mechanism of the synthesized edge-enriched WS_2_ can be described via two different reactions: (i) chemisorption and (ii) physisorption, as shown in Figure 7. The first one lies in the adsorption/desorption between the adsorbed oxygen species at the material active sites and the target gas.^58^ Herein, oxygen species cannot be neglected, as the sensor was flushed with synthetic air to clean the material surface and reach a steady state. Therefore, when the sensor is exposed to dry synthetic air, the oxygen molecules interact with the WS_2_ surface and get adsorbed in the form of O_2_^–^,^65,66^ leading to the extraction of electrons from the WS_2_ valence band and the formation of a hole accumulation layer (HAL) at the same band.^30,67^ It is worth mentioning that the adsorbed oxygen species depend on the sensor working temperature since they can be adsorbed in the form of O_2_^–^ (<150 °C), O^–^(150 to 400 °C), or O^2–^(>400 °C).^58^

The following equation can represent this chemical reaction:
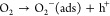
9When the sensing layer is exposed to the NH_3_ environment, the resistance increases due to the electron donor nature of NH_3_ (presence of a lone pair of electrons on it), which results in a decrease in the concentration of holes in the HAL region and confirms the p-type semiconductor behavior of WS_2_. The following equation can explain this reaction:^68^
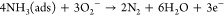
10In physisorption reaction, NH_3_ molecules can seep into the layers of WS_2_ and get easily adsorbed onto the edge sites of WS_2_, thanks to the strong electronegativity of the sulfur layer in WS_2_. Hence fore, it injects electrons into the WS_2_ layers and itself gets converted into NH_4_^+^. The electrons released on the WS_2_ valence band combine with the existing holes, thereby decreasing the space charge layer, reducing the concentration of holes, and increasing the material resistance.^69^ Our first-principles calculations back up these findings. We found no evidence of any hybridization and a very small charge transfer, suggesting the absence of chemical bonding between NH_3_ and WS_2_. The close resemblance of the conduction and valence states near the Fermi level in the combined system to their isolated counterparts, coupled with the low adsorption energy and minimal charge transfer, further suggests that the interaction between WS_2_ and NH_3_ is predominantly attributed to physisorption. Nevertheless, despite the physisorption evidence found in our DFT results, it is essential to acknowledge that the chemisorption process cannot be dismissed, as mentioned earlier in this section.

## Conclusions

A facile, scalable, and high-yield atmospheric pressure chemical vapor deposition (APCVD) technique was developed to synthesize plate-shaped, edge-enriched tungsten disulfide (WS_2_) powder from commercial tungsten oxide (WO_3_) precursor powders. Thin films of the synthesized WS_2_ were deposited on alumina transducer substrates using a simple airbrushing deposition method to be used as sensing material. The WS_2_ sensors exhibited excellent sensitivity toward ammonia (NH_3_) at a moderate operating temperature of 150 °C. This represents the first demonstration of a high-yielding synthesis technique that produces WS_2_ powder, which can be readily deposited on a wide range of substrate materials. The cross-sensitivity of the sensors was evaluated toward potential interfering gases, including H_2_, benzene, CO, and NO_2_. The sensors showed negligible or small responses to these species, with the maximum response for NH_3_. First-principles calculations were performed to confirm that the interaction between the WS_2_ and NH_3_ is predominantly physisorption-based. Furthermore, the impact of a 50% relative humidity background was to be limited, resulting in only a slight decrease in sensor response. These findings confirm the promising gas sensing characteristics of the edge-enriched WS_2_-based sensors for the selective detection of low concentrations of NH_3_ under realistic operating conditions at moderate temperatures. The development of this facile, scalable, and high-yield APCVD synthesis technique for WS_2_ powder, coupled with the demonstrated gas sensing performance, opens new avenues for the practical implementation of WS_2_-based sensors for real-world applications.

## Supporting Information Available

Schematics of APCVD, air brushing, synthesis mechanism, and cross-section of airbrushed film; FESEM of as-synthesized WS_2_; XRD results; humidity results; selectivity data; comparison table with literature (PDF)
