# Supplementary material for: High-Yield WS2 Synthesis through Sulfurization in Custom-Modified Atmospheric Pressure Chemical Vapor Deposition Reactor, Paving the Way for Selective NH3 Vapor Detection
Source: ACS Appl Mater Interfaces. 2024 Sep 2;16(36):48585–97. doi: 10.1021/acsami.4c10077 (PMC11403549; doi:10.1021/acsami.4c10077)
Supplement: Supplementary file 1 — am4c10077_si_001.pdf [file am4c10077_si_001.pdf]

Supporting information

High-Yield WS_2 Synthesis through Sulfurization in Custom-Modified APCVD Reactor, Paving the Way for Selective NH_3 Vapor Detection

Shuja Bashir Malik[1,2,3]*, Fatima Ezahra Annanouch*[1,2,3*]*, Ransell D´Souza*[5]*, Carla Bittencourt*[4]*, Milica Todorović*[5,*]*, Eduard Llobet*[1,2,3]

1 Universitat Rovira i Virgili, MINOS, Països Catalans 26, 43007 Tarragona, Catalunya, Spain.
2 IU-RESCAT, Research Institute in Sustainability, Climatic Change and Energy Transition, Universitat Rovira i Virgili, Joanot Martorell 15, 43480 Vila-seca, Spain.
3 TecnATox - Centre for Environmental, Food and Toxicological Technology, Universitat Rovira i Virgili, Avda. Països Catalans 26, 43007 Tarragona, Spain
4 Chimie des Interactions Plasma-Surface (ChIPS), Research Institute for Materials Science and Engineering, University of Mons, 7000 Mons, Belgium
5 Department of Mechanical and Materials Engineering, Faculty of Technology, University of Turku, Vesilinnantie 5, 20500, Turku, Finland
* Correspondence: authors' email: fatimaezahra.annanouch@urv.cat and milica.todorovic@utu.fi

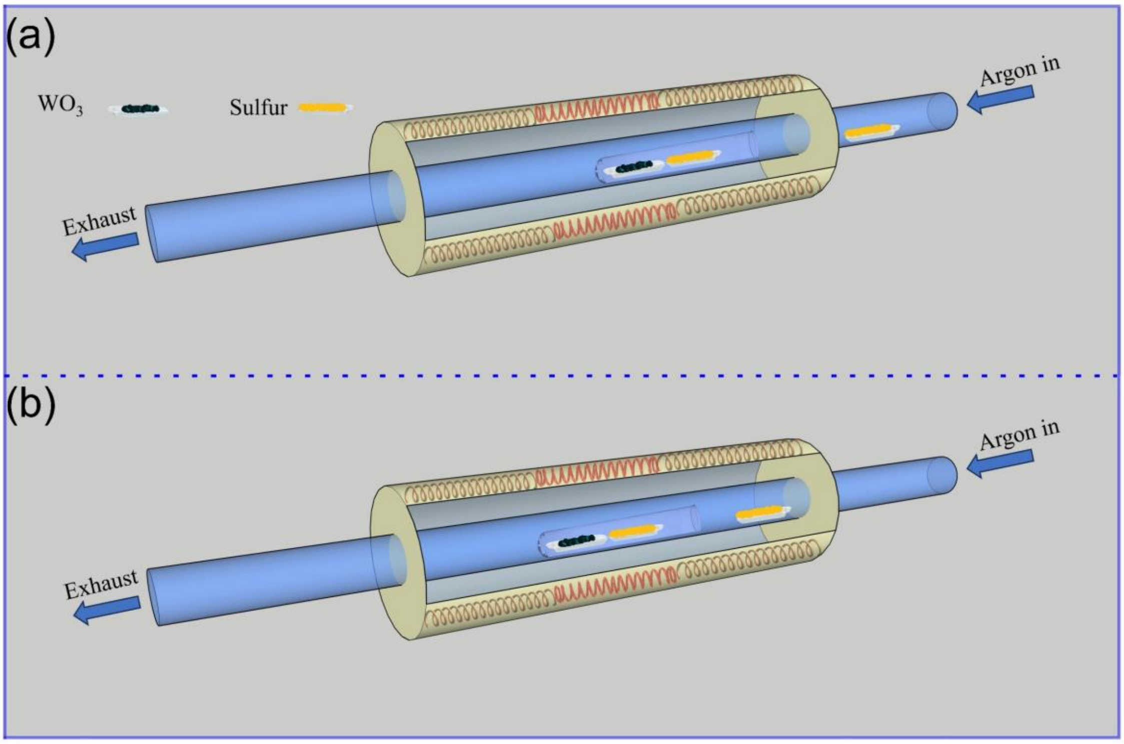

Figure S1: Schematic of the APCVD furnace used for WS_2 synthesis, illustrating the strategic placement of the precursor (WO_3) and sulfur boats. (a) Secondary sulfur boat positioned outside the furnace upstream of Ar flow. (b) After the furnace temperature reaches 900°C, the external quartz tube is adjusted to place the secondary boat in the 400°C zone of the furnace.

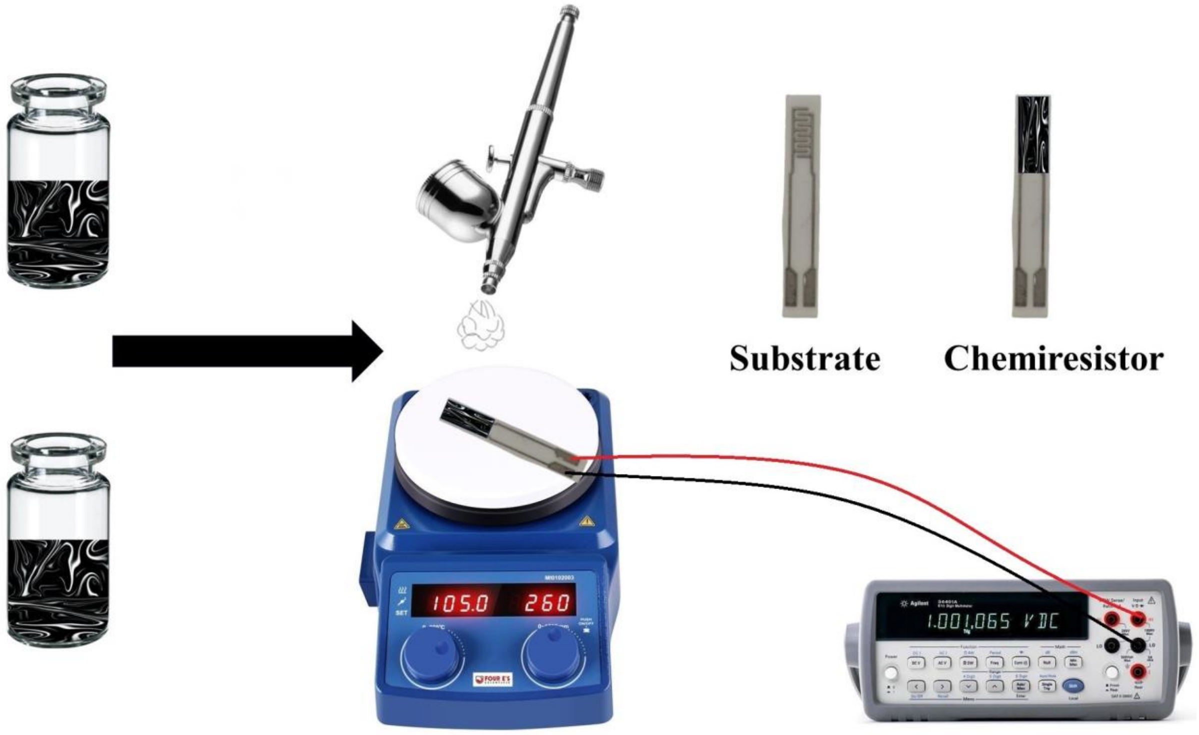

Figure S2: Homemade airbrushing setup.

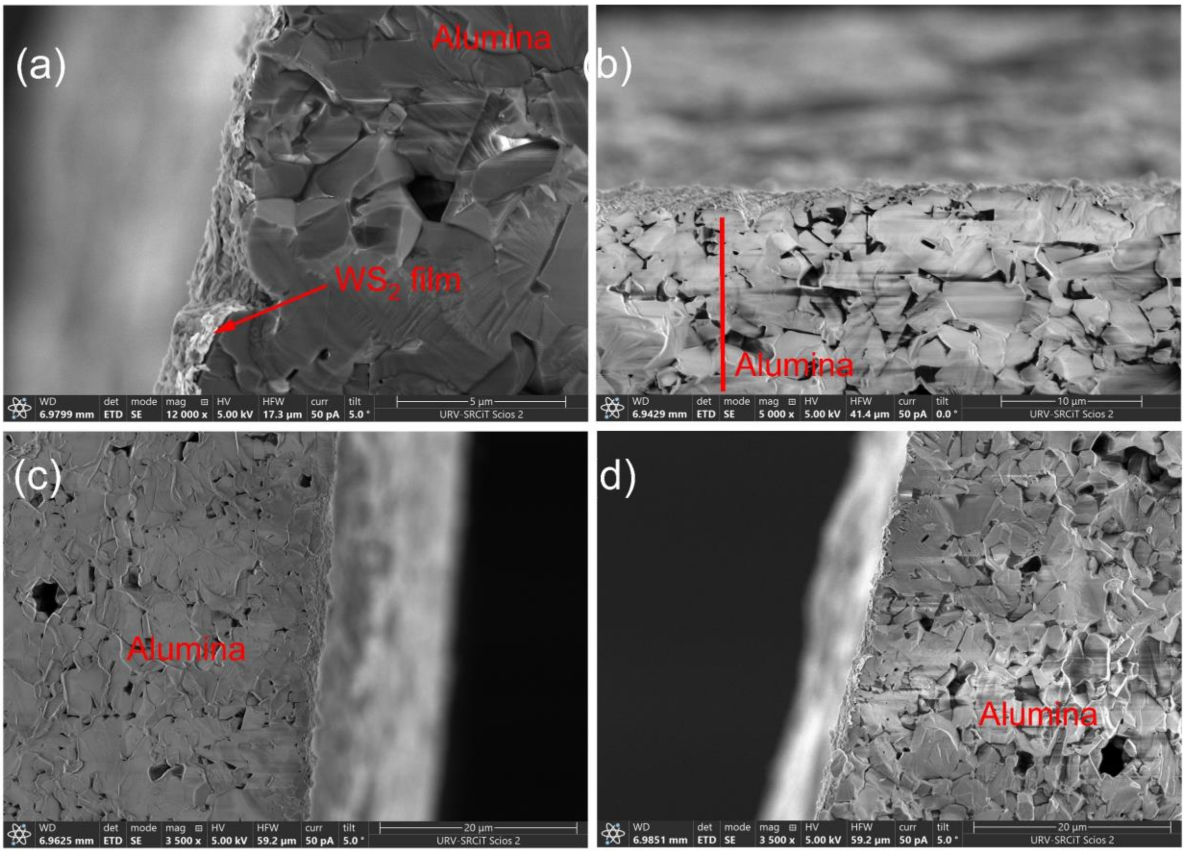

Figure S3: (a-d) Cross section FESEM images of alumina substrate with WS_2 film deposited via airbrushing on alumina substrate.

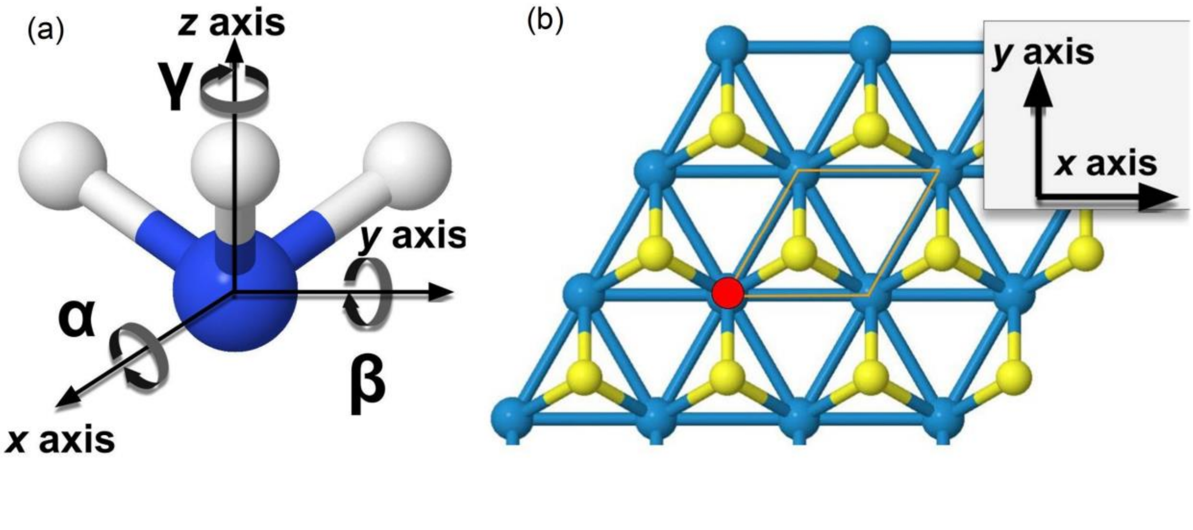

Figure S4. Translational and rotational degrees of freedom for the adsorption structure search: a) side view and top view illustration of the NH_3 molecular rotations implemented within the internal molecular frame of reference; (b) illustration of the translational motion of the molecule above the WS_2 substrate. The orange line delineates the periodic search region.

Direction [001] is perpendicular to the [100]-[010] plane of the substrate, and the red point denotes the centroid of the NH_3 molecule.

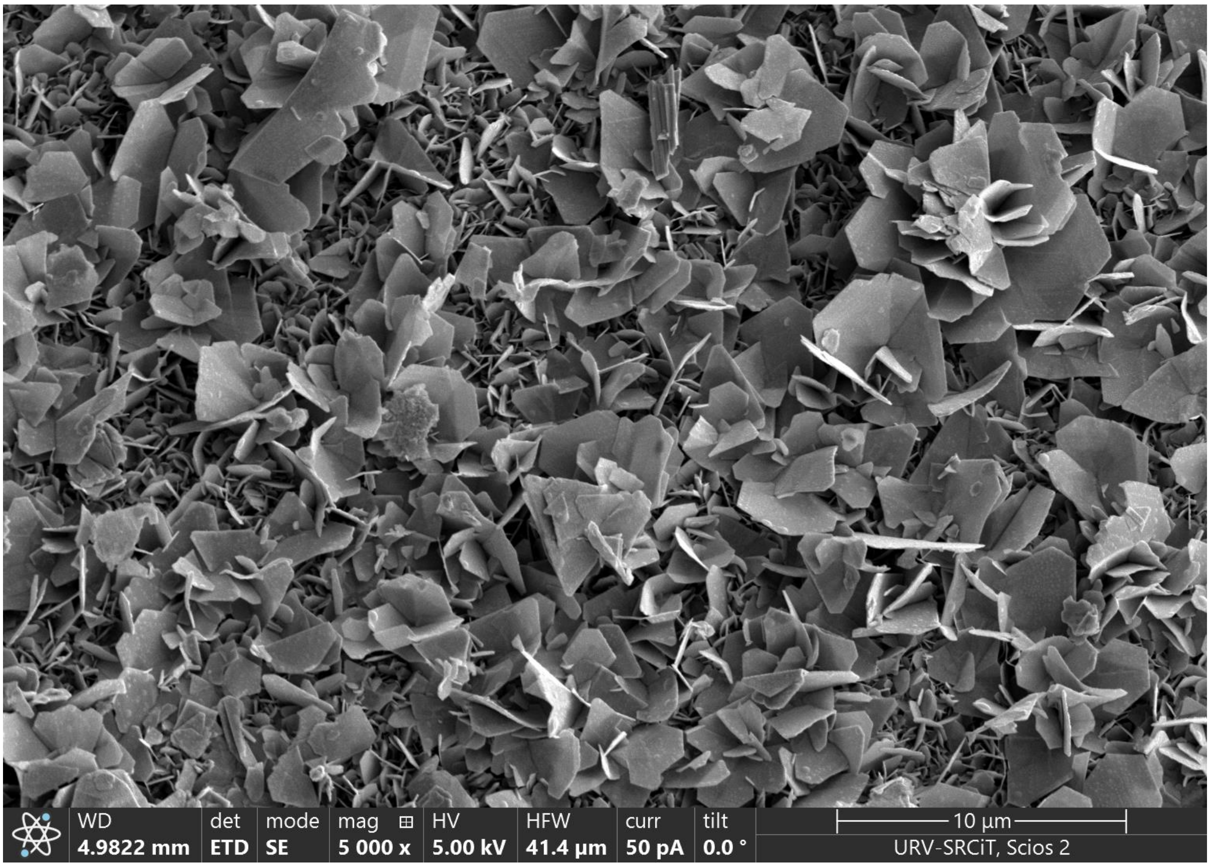

Figure S5: FESEM image of as synthesized WS_2.

Figure S6 shows the XRD diffractogram recorded on the as-synthesized WS_2 in the range of $2\theta = 5^o$ to 60°. The diffraction peaks match with a rhombohedral phase of WS_2 (ICDD card number: 84-1399) with lattice constants a= 0.3158 nm and c= 1.849 nm belonging to the R3m space group. The XRD pattern reveals the presence of intense peaks at 14°, 29°, 33°, 44° and 60°, in agreement with the (003), (006), (101), (009), and (0012) crystal planes of rhombohedral WS_2. Additionally, small peaks were also detected at 23°, 24°, and 25°, which were indexed to (002), (020), and (200) crystal planes of triclinic WO_3 traces (ICDD card number: 32-1395) belonging to space group P-1(2) with lattice constants a= 0.7309 nm, b= 0.7522 nm, and c= 0.7678 nm.

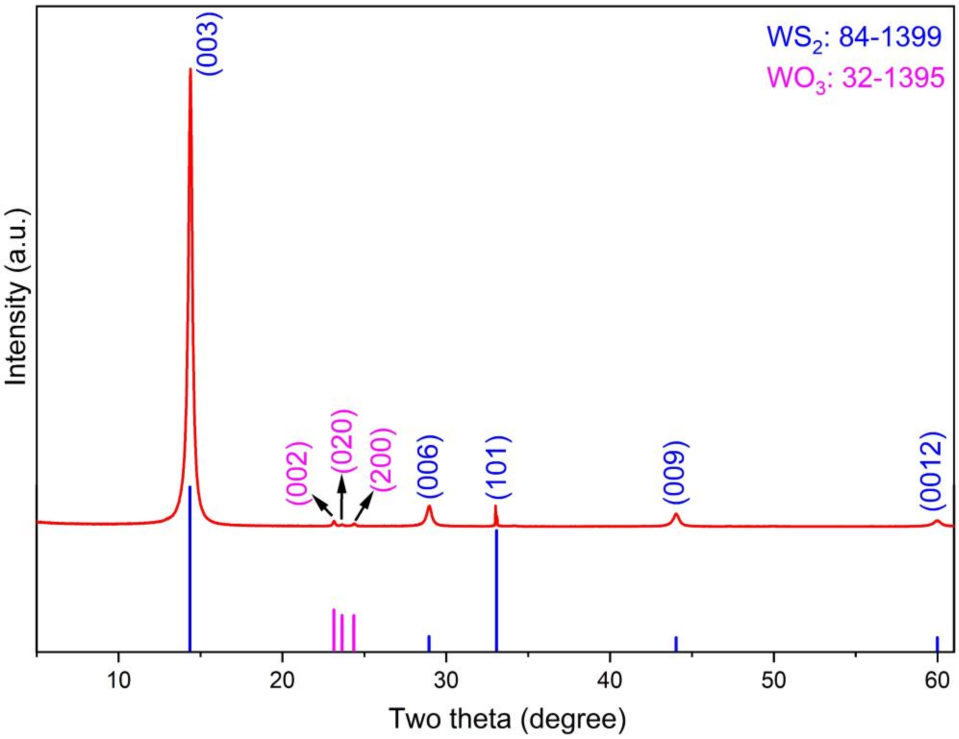

Figure S6: XRD diffractogram of as-synthesized WS_2 airbrushed on a silicon oxide substrate.

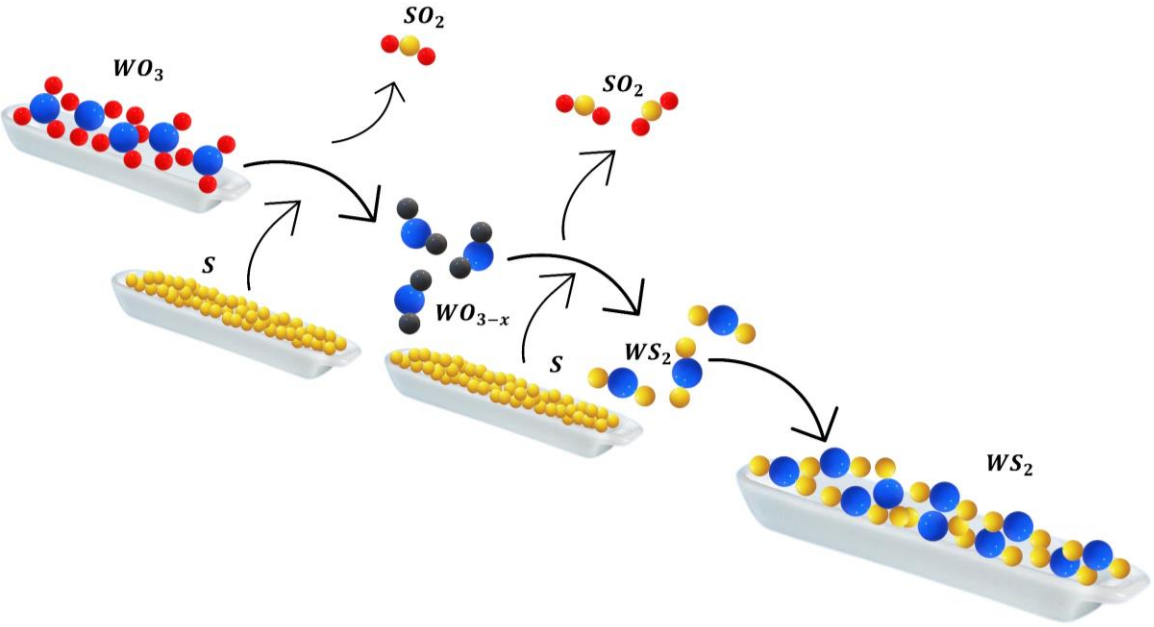

Figure S7. Synthesis mechanism of edge enriched WS_2 powder.

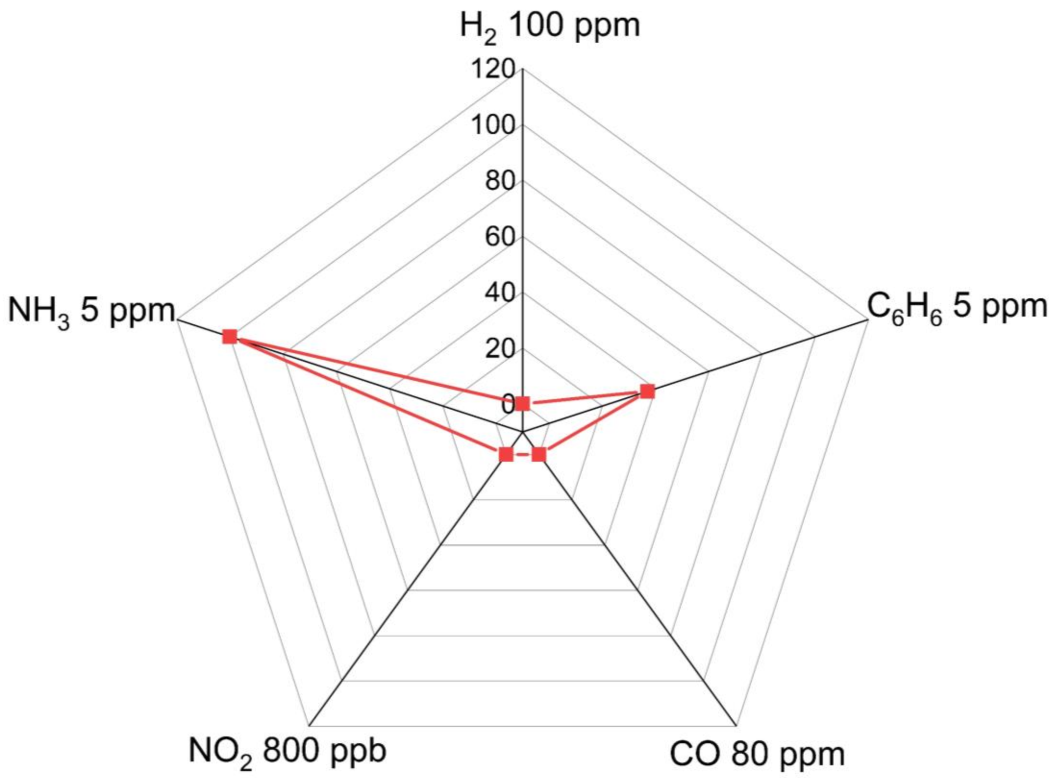

Figure S8: Radar plot of the response of WS_2 towards NO_2 (800 ppb), H_2 (100 ppm), carbon monoxide (80 ppm), ammonia (5 ppm), and benzene (5 ppm) at 150ºC.

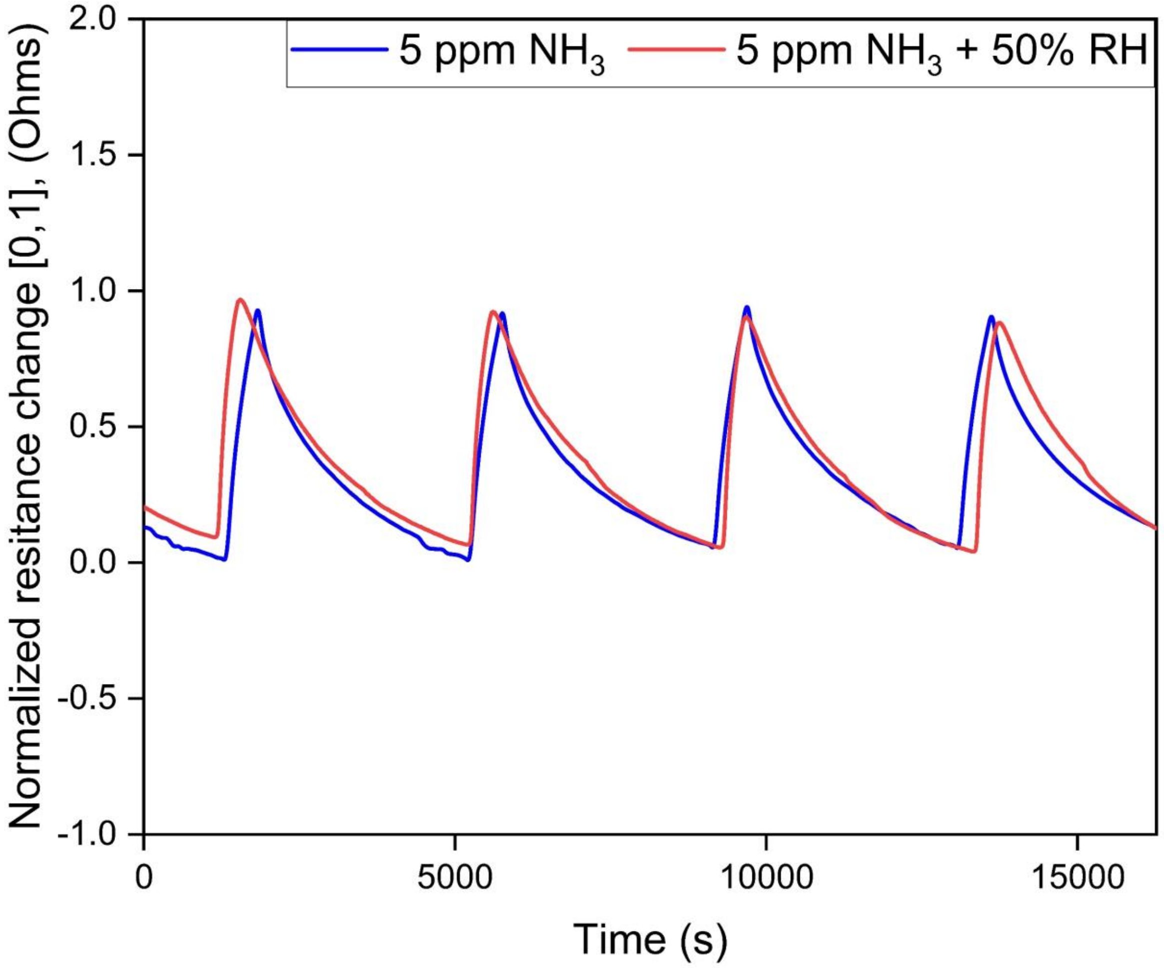

Figure S9. Dry and relative humidity cross-sensitivity to 5 ppm NH_3 at 150°C. The sensor resistance changes are normalized to [0,1].

Table S1: Ammonia gas sensing characteristics reported in this work are compared with various TMD Materials.

2D material	Conc. Studied (ppm)	Working temp. (ºC)	Response	LoD (ppm) experimental	Chamber volume (mL)	Selectivity	Ref.
WSe_2 NFs	40	150	24.65%	2	35	NO_2, C_6H_6, CO and H_2	1
PPy/WS_2	200	28	30.10%	50	1547	H_2, EtOH, CO, CO_2	2
rGO/WS_2	50	33.5	121%	10	-	NO_2, acetone, EtOH, formaldehyde, methanol, C_6H_6	3
MoS_2 nanosheets	10	100	30%	2	35	CO, H_2, NO_2	4
WS_2	100	35	4.61%	50	-	H_2S, SO_2, acetone, CO	5
Pt Qd/WS_2	500	25	14.5%	50	-	EtOH, acetic acid, CO, H_2, NO_2, ethyl acetate, n-propanol	6
WS_2	5	150<br>100<br>25	100%<br>79%<br>1%	<1	35	C_6H_6, H_2, NO_2, CO	This work

References

(1) Alagh, A.; Annanouch, F. E.; Sierra-Castillo, A.; Haye, E.; Colomer, J.-F.; Llobet, E. Three-Dimensional Assemblies of Edge-Enriched WSe 2 Nanoflowers for Selectively Detecting Ammonia or Nitrogen Dioxide. *ACS Appl. Mater. Interfaces* **2022**, *14* (49), 54946–54960. https://doi.org/10.1021/acsami.2c16299.

(2) Sood, Y.; Lawaniya, S. D.; Mudila, H.; Awasthi, K.; Kumar, A. Polypyrrole-Tungsten Disulphide 2D Nanocomposites for Ammonia Sensing. *Sensors Actuators B Chem.* **2023**, *394* (June), 134298. https://doi.org/10.1016/j.snb.2023.134298.

(3) Wang, X.; Gu, D.; Li, X.; Lin, S.; Zhao, S.; Rumyantseva, M. N.; Gaskov, A. M. Reduced Graphene Oxide Hybridized with WS2 Nanoflakes Based Heterojunctions for Selective Ammonia Sensors at Room Temperature. *Sensors Actuators, B Chem.* **2019**, *282* (November 2018), 290–299. https://doi.org/10.1016/j.snb.2018.11.080.

(4) Annanouch, F. E.; Alagh, A.; Umek, P.; Casanova-Chafer, J.; Bittencourt, C.; Llobet, E. Controlled Growth of 3D Assemblies of Edge Enriched Multilayer MoS2 Nanosheets for Dually Selective NH3 and NO2 Gas Sensors. *J. Mater. Chem. C* **2022**, *10* (30), 11027–11039. https://doi.org/10.1039/d2tc00759b.

(5) Qin, Z.; Song, X.; Wang, J.; Li, X.; Wu, C.; Wang, X.; Yin, X.; Zeng, D. Development of Flexible Paper Substrate Sensor Based on 2D WS2 with S Defects for Room-Temperature NH3 Gas Sensing. *Appl. Surf. Sci.* **2022**, *573* (September 2021), 151535. https://doi.org/10.1016/j.apsusc.2021.151535.

(6) Ouyang, C.; Chen, Y.; Qin, Z.; Zeng, D.; Zhang, J.; Wang, H.; Xie, C. Two-Dimensional WS 2 -Based Nanosheets Modified by Pt Quantum Dots for Enhanced Room-Temperature NH 3 Sensing Properties. *Appl. Surf. Sci.* **2018**, *455* (April), 45–52. https://doi.org/10.1016/j.apsusc.2018.05.148.
